# Labour market strategies addressing precarious employment and its impacts: A systematic review

**DOI:** 10.1093/eurpub/ckac129.096

**Published:** 2022-10-25

**Authors:** V Gunn, EF Vignola, DH Wegman, C Hogstedt, T Bodin, C Orellana, P O'Campo, M Albin, C Håkansta, PWR Consortium

**Affiliations:** 1 Unit of Occupational Medicine, Institute of Environmental Medicine KI, Stockholm, Sweden; 2 MAP Centre for Urban Health Solutions, Li Ka Shing Knowledge Institute, Toronto, Canada; 3 Lawrence S. Bloomberg Faculty of Nursing, University of Toronto, Toronto, Canada; 4 Community Health and Social Sciences, Graduate School of Public Health and Health Policy, City University NY, New York, USA; 5 University of Massachusetts Lowell, Lowell, USA; 6 La Isla Network, Washington, USA; 7 Center for Occupational and Environmental Medicine, Stockholm Region, Stockholm, Sweden; 8 Dalla Lana School of Public Health, University of Toronto, Toronto, Canada; 9 Working Life Science, Business School, Karlstad University, Karlstad, Sweden

## Abstract

**Background:**

Precarious employment (PE), characterized by reduced worker rights, and employment and income insecurity, has complex public health implications including negative impacts on workers’ mental and physical health, occupational health and safety, wellbeing, and inequities in access to health and social protections. There is, however, a knowledge gap regarding effectiveness of interventions. We describe findings from a review of evaluated interventions with potential to address PE.

**Methods:**

Our systematic review followed the 2020 PRISMA framework and covered PubMed, Scopus, Web of Science, and sources of grey literature. We included qualitative, quantitative, or mixed-methods studies evaluating initiatives to reduce workers’ PE published from 2000 to 2021 and focused on adult workers.

**Results:**

The 23 eligible studies from across the world evaluated diverse strategies addressing PE including tax and trade reforms, industrial disputes legislation, business registration, and use of incentives to stimulate permanent contracts. Also included were union strategies to reach precarious workers, the provision of social benefits, and youth apprenticeships. Generally, while most initiatives had the potential to tackle certain PE aspects, they usually acted only on one or two PE dimensions. Additionally, the evaluation components were missing key details, thus, limiting the generalizability of findings, as did the heterogeneity of study designs, initiative purposes, economic and political context, and diverse populations targeted.

**Conclusions:**

The increase in PE prevalence and its complex health implications requires sustainable upstream public health solutions. Multidisciplinary collaborations among public health and occupational health practitioners along with researchers, evaluation specialists, economists, and politicians could facilitate the implementation and evaluation of policies and standards regulating and monitoring PE and its health impacts.

**Key messages:**

• Precarious employment has complex public health implications.

• Sustainable solutions to address precarious employment must be upstream and multidisciplinary.

